# Advances and challenges of endometrial organoids in modeling endometrial function: basic, clinical, and future perspectives

**DOI:** 10.3389/fbioe.2026.1910098

**Published:** 2026-09-28

**Authors:** Jiwen Tian, Jie Yang, Huawei Wang, Zhenfang Su, Tianqing Li, Li Tang

**Affiliations:** 1 Department of Reproduction and Genetics, The First Affiliated Hospital of Kunming Medical University, Kunming, Yunnan, China; 2 State Key Laboratory of Primate Biomedical Research, Institute of Primate Translational Medicine, Kunming University of Science and Technology, Kunming, Yunnan, China

**Keywords:** embryo implantation, endometrial function, endometrium, maternal-fetal interface, organoids, reproductive medicine

## Abstract

As robust *in vitro* models that faithfully replicate the physiological functions of the human endometrium, endometrial organoids have emerged as pivotal tools for deciphering the regulatory mechanisms of endometrial receptivity and disease pathogenesis. Currently, insufficient vascularization and immunocompetence, phenotypic drift during long-term culture, and the absence of standardized protocols for clinical translation remain major challenges in endometrial organoid research. These limitations constrain their broader application in elucidating embryonic implantation mechanisms and advancing personalized therapeutics. In this review, we mainly introduce the recent progress in the endometrial organoids and their utility in studying endometrial development, decidualization regulation, and endometrial pathologies. Furthermore, we highlight the potential applications in reproductive medicine. Finally, we discussed the current challenges and future perspectives of endometrial organoid and its interdisciplinary integration. We anticipate that their translational potential will revolutionize the studies on uncovering the mechanisms of early implantation failure to inform diagnosis and therapeutics.

## 1 Introduction

Compared to other mammalian species, humans exhibit remarkably low reproductive efficiency. Among women with reproductive age, the probability of natural conception per menstrual cycle is as low as 20%–30% (Zinaman et al., 2019). Strikingly, nearly 70% of conceptions arrest in development prior to reaching viability, with over 50% of pregnancies lost in the preclinical stage primarily due to implantation failure or chemical pregnancy (Shaulov et al., 2020). Human embryo implantation is a highly specialized process, critically dependent on the cyclic regulation of the endometrium to orchestrate the periodic opening of the window of implantation (WOI). Consequently, aberrant endometrial development or dysfunction frequently results in embryo implantation failure or impaired placentation. These defects not only compromise overall pregnancy outcomes but also precipitate human-specific obstetric complications, such as fetal growth restriction (FGR) and preeclampsia (Kadam et al., 2019; Norwitz, 2006). Furthermore, the adverse effects of these placental defects can persist well beyond birth, predisposing offspring to abnormal long-term growth and developmental trajectories (Sun et al., 2020).

The inefficiency of human embryo implantation and the manifestation of certain human-specific pregnancy complications are fundamentally dictated by the complex crosstalk at the maternal-fetal interface. The unique process of human decidualization necessitates distinct implantation strategies, modes of maternal-fetal interaction, and diverse mechanisms of placentation compared to other species (Enders and Schlafke, 1978). Uterine development spans the entire human fetal period and continues through the end of puberty. Consequently, early impairments in endometrial development or disease-induced functional abnormalities ultimately disrupt the critical interactions between embryonic trophectoderm cells and endometrial cells. As the initiating event of pregnancy, implantation requires trophoblast cells to firmly adhere to the luminal epithelium during the open WOI, followed by invasion into the endometrial stroma to facilitate progressive placental development. However, because the precise preparatory events and the detailed real-time steps of early *in vivo* implantation cannot be directly observed in humans, our understanding of this process remains rudimentary. Furthermore, animal models alone are insufficient to fully recapitulate the unique specificity and complexity of human implantation regulation.

Currently, the knowledge of human implantation relies heavily on *in vitro* models. While continuous advancements in laboratory techniques and the refinement of *in vitro* systems have provided valuable insights into embryo implantation, these models still exhibit inherent limitations in accurately simulating the authentic physiological microenvironment of the endometrium and capturing the full complexity of the implantation process (Ojosnegros et al., 2021). By integrating the developmental and anatomical characteristics of human endometrial tissue, its cyclical functional changes, the complex regulation of the WOI, and the cascade of events triggered by trophectoderm attachment to the uterine luminal epithelium, this review aims to systematically explore the research value of endometrial organoids. Specifically, we will discuss their utility in studying endometrial development, decidualization regulation, and endometrial pathologies, as well as their translational potential in uncovering the mechanisms of early implantation failure to inform diagnosis and therapeutics.

In recent years, despite continuous advancements in ART, particularly the significant progress made in optimizing pre-implantation embryo development, the overall pregnancy success rate remains plateaued at approximately 55%–60%. This is largely attributed to recurrent implantation failure (RIF), which constitutes a major cause of preclinical pregnancy loss (Shaulov et al., 2020). Endometrial organoids hold immense potential for future research. It is imperative to explore strategies for constructing organoid models that more faithfully simulate the maternal-fetal interface during implantation. Leveraging these advanced models will enable us to decipher the mechanisms underlying the establishment of endometrial receptivity, comprehensively map the regulatory networks governing embryo-endometrial crosstalk, and enhance the prevention and management of pregnancy complications. Ultimately, this will provide a robust theoretical foundation for improving ART outcomes and developing personalized medicine approaches for pregnancy-related disorders.

## 2 The evolution of endometrial organoid development

Organoids are three-dimensional (3D) *in vitro* models formed through cellular self-organization that faithfully recapitulate the structural and functional characteristics of their native tissues. Since the landmark derivation of *in vitro* organoids from mouse intestinal stem cells by Dutch researchers in 2009 (Sato et al., 2009), organoid technology has rapidly evolved and garnered widespread scientific interest. Currently, disease modeling and drug screening rely predominantly on traditional cell lines and animal models. However, conventional two-dimensional (2D) cell lines inherently fail to reproduce the complex 3D architecture of *in vivo* tissues. Conversely, while animal models can simulate physiological microenvironments, they are labor-intensive, time-consuming, and cost-prohibitive. Furthermore, due to significant interspecies disparities, the extent to which animal models can accurately reflect human tissue functions and human-specific pathological states remains a subject of ongoing validation.

Organoids are emerging as a novel experimental system distinct from both traditional cell lines and animal models. They not only partially mimic the 3D structural complexity of tissues *in vitro* but also retain the practical advantages of cell lines with scalability for serial passaging. Moreover, this technology facilitates the establishment of complex, multi-organ crosstalk models (Rossi et al., 2018). Consequently, organoids hold tremendous translational potential across a broad spectrum of fields, including basic medical research, disease modeling for targeted drug screening, toxicology, oncology, microbiology, and regenerative medicine.

### 2.1 Diversity of sources and *in vitro* culture systems for endometrial organoids

The genetic background of immortalized or tumorigenic cell lines, such as Ishikawa cells, differs substantially from that of the native *in vivo* epithelium. Conversely, primary endometrial epithelial cells are notoriously difficult to expand long-term while maintaining their phenotypes *in vitro*, and traditional two-dimensional (2D) cell cultures intrinsically fail to recapitulate the authentic 3D architectural complexity of the native tissue. Consequently, in recent years, substantial research efforts have been directed toward the development of endometrial organoids.

Boretto et al. and Turco et al. established endometrial organoids for the first time in 2017, specifically glandular epithelial organoids derived from both mice and humans (Turco et al., 2017; Boretto et al., 2017). In these foundational studies, researchers enzymatically dissociated primary endometrial biopsy samples, resuspended the cells in 70% Matrigel droplets, and formulated a defined, complex culture medium capable of promoting the formation and maintenance of organoids derived from primary tissues or pluripotent stem cells. This optimized medium was supplemented with WNT signaling activators (WNT ligands and R-spondin1), growth factors (EGF, FGF10), a TGF-β inhibitor (A83-01), a BMP inhibitor (Noggin), and nicotinamide.

Importantly, these organoids can be established not only from endometrial biopsies taken throughout the whole menstrual cycle but also from the postpartum decidua of patients with known pregnancy outcomes (e.g., preterm or term placentas) (Marinić et al., 2020), as well as from the endometrium of postmenopausal women (Turco et al., 2017). Furthermore, recent advancements have enabled the non-invasive isolation of viable endometrial tissue fragments from menstrual blood for successful organoid generation (Cindrova-Davies et al., 2021). Glandular epithelial organoids derived via these diverse ways can all be subjected to long-term passaging while maintaining remarkable genetic and phenotypic stability. Notably, upon hormonal induction, these *in vitro* models demonstrate the capacity to differentiate into the two primary epithelial subtypes found *in vivo*: secretory cells expressing progestogen-associated endometrial protein (PAEP), and ciliated cells positive for acetylated α-tubulin (Turco et al., 2017; Boretto et al., 2017).

### 2.2 Refinement and limitations of endometrial assembloids

To simulate *in vivo* interactions between different tissues and organs, researchers have recently achieved direct physical contact between distinct organoids via 3D co-culture systems. These fused organoids, termed “assembloids,” facilitate intercellular crosstalk and drive the emergence of novel structural and functional characteristics (Koike et al., 2019). Building upon the establishment of early endometrial glandular epithelial organoids, Cheung et al. co-cultured human pluripotent stem cell-derived endometrial stromal fibroblasts with glandular epithelial organoids in Matrigel, demonstrating their capacity to differentiate into decidual cells in response to hormones (Cheung et al., 2021). Conversely, Rawlings et al. incorporated primary endometrial stromal cells into glandular epithelial organoids to investigate the senescence phenotype of decidual cells following hormonal stimulation, exploring its implications in the menstrual cycle and the promotion of pregnancy (Rawlings et al., 2021). Rawlings et al. subsequently established a standardized methodological framework for assembloid generation, differentiation, and embryo co-culture, providing a reproducible platform for studying implantation (Rawlings et al., 2024). Through a series of experiments, Tian et al. evaluated the proliferative viability and physiological functionalities of assembloids constructed with varying epithelial-to-stromal cell ratios. By identifying the optimal cellular composition, the research revealed that intercellular crosstalk within the endometrium dictates its unique physiological functions (Tian et al., 2023).

A recent study generated highly complex assembloids by mixing all single cells and cell clusters derived from native endometrial tissue, thereby incorporating resident immune and endothelial cells (Zhang et al., 2023). Transcriptomic analysis revealed that ciliated epithelial cells, in coordination with immune cells and secretory epithelium, regulate angiogenesis via the Nrp1-VEGFA and NRP2. This study successfully mapped the intricate intercellular crosstalk characteristic of the window of implantation (WOI) *in vitro*. However, a notable limitation of these assembloids is the low abundance of immune cells, predominantly restricted to T cells expressing TSPYL2, RSRP1, and CD44, while lacking other crucial cell types involved in immune homeostasis (Zhang et al., 2023). This raises critical questions regarding whether current culture systems are adequate to support the growth and development of every cellular subtype. Furthermore, it warrants investigation into whether specific cell populations are progressively lost after multiple passages, consequently impairing the model’s ability to fully recapitulate the tissue phenotype.

In another study, human umbilical vein endothelial cells (HUVECs) were introduced into endometrial assembloids (Shibata et al., 2024). After 4 days, the HUVECs interconnected to form luminal structures and an endothelial network. The presence of HUVECs was found to inhibit apoptosis in both epithelial and stromal cells. Moreover, snRNA-seq data analysis demonstrated extensive interactions between the epithelium, endothelium, and stroma. Epithelial-derived ligands—such as vascular endothelial growth factor A (VEGFA), semaphorin 3C (SEMA3C), and platelet-derived growth factor C (PDGFC)—likely target corresponding receptors in endothelial and stromal cells, including fms-like tyrosine kinase 1 (FLT1), platelet-derived growth factor receptor alpha (PDGFRA), neuropilin-1 (NRP1), plexin D1 (PLXND1), and EGF receptor (EGFR). The breakthrough of the study is elucidation of the interactions between epithelial, endothelial, and stromal cells within endometrial assembloids. Nonetheless, further evidence is required to substantiate the functional and phenotypic equivalence between HUVECs and native endometrial microvascular endothelial cells. Additionally, given that the regulation of blood flow is the primary function of the *in vivo* vasculature, engineering a truly functional, perfusable vascular network remains a formidable challenge for future research.

Ren et al. employed CODA-based 3D tissue mapping to design multi-compartment assembloids with spatially distinct epithelial (Matrigel) and stromal (collagen I) compartments, recapitulating all three phases of the menstrual cycle (Ren et al., 2025). Co-culture of assembloids with trophoblast spheroids has further been validated to support EVT migration and invasion with invadopodia formation (Handzhiyska et al., 2025). Despite these advances, stromal and immune cells are difficult to maintain through passaging, and systemic hormonal regulation cannot be fully recapitulated. Future refinements, including the addition of immune cells, endothelial cells, and microfluidic systems, will enable more comprehensive modeling of the endometrial microenvironment and embryo implantation.

### 2.3 Apical-out organoid techniques

The uterine epithelium comprises a single layer of hormone-responsive polarized epithelial cells that line the lumen and form tubular glands. Endometrial epithelial organoids can recapitulate cell composition and hormone responses *in vitro*. However, a major limitation of the use of endometrial epithelial organoids cultured in Matrigel and other hydrogels is the inner location of the apical membrane (apical-in organoid), thereby hindering direct access to the apical surface of the epithelium to study interactions with the embryo or infectious agents such as viruses and bacteria.

To overcome this limitation, Ahmad et al. developed a suspension culture method to reverse the polarity of human endometrial epithelial organoids (Ahmad et al., 2024). The resulting apical-out organoids preserve apical-basolateral orientation and remain fully responsive to ovarian steroid hormones. Notably, progesterone treatment induces secretory transformation, including decreased microvilli and cilia and increased secretory granules. Shibata et al. further advanced this approach by employing a collagen-Matrigel mixed matrix to generate hormone-responsive apical-out endometrial organoids that emulate the *in vivo* architecture of endometrial tissue, featuring exposed apical epithelium (Shibata et al., 2024). When co-cultured with human blastoids, this maternal-fetal assembloid system recapitulates critical implantation stages. Fujimura et al. also established an apical-out mouse endometrial organoid incorporating both epithelial and stromal cells, and demonstrated its utility in recapitulating implantation with mouse blastocysts (Fujimura et al., 2025). Guo et al. extended this approach to ruminants by establishing the first sheep endometrial luminal epithelial organoids, which were generated from caruncular tissues. These organoids successfully achieved apical-out polarity through suspension culture. When co-cultured with trophoblast cells or blastocysts, apical-in organoid demonstrated the capacity to support embryonic development and trophoblast proliferation (Guo et al., 2026). Beyond implantation studies, another research utilized apical-out endometrial organoids to model pathogen-induced endometritis via natural infection routes, highlighting the broader applicability of this technology (Zhang et al., 2024). Unlike conventional apical-in organoids, this novel model provide direct accessibility and a more faithful recapitulation of the *in vivo* epithelial orientation for implantation and reproductive disease studies.

### 2.4 Further optimizations in the extracellular matrix (ECM)

Currently, organoid culture predominantly relies on hydrogel-based systems (Figure 1). Adult stem cells with regenerative capacity, derived from various tissues and organs, are embedded within a matrix to simulate the native extracellular scaffolding of adult tissues (Rossi et al., 2018). The extracellular matrix (ECM) serves not merely as a structural scaffold for cell growth; it actively orchestrates organoid polarity and architecture by spatially restricting the diffusion and accessibility of intercellular signaling molecules. Consequently, the ECM itself acts as a critical determinant of tissue specificity (Lu et al., 2012). In the context of endometrial organoids, the widely utilized ECM is primarily Matrigel, a basement membrane extract purified from mouse sarcomas (Boretto et al., 2017; Turco et al., 2017; Cheung et al., 2021; Garcia-Alonso et al., 2021). When encapsulated within Matrigel droplets, endometrial glandular fragments spontaneously self-organize into luminal structures (Figure 1). While highly efficient in promoting organoid growth and self-organization, Matrigel possesses inherent limitations, notably its animal origin and undefined, highly variable composition. Beyond the ethical and biosafety concerns that limit its application in regenerative medicine, its physicochemical properties diverge significantly from the native human endometrial ECM. To better approximate the ECM characteristics of the mid-luteal phase endometrium, Rawlings et al. supplemented Matrigel with 3% collagen I for the co-culture of endometrial assembloids (Rawlings et al., 2021). Similarly, Tian et al. incorporated collagen I at a 1:1 ratio with Matrigel (Figure 1). They observed that the resulting hybrid matrix exhibited a Young’s modulus comparable to that of native endometrial tissue, thereby mimicking *in vivo* physical stiffness and significantly enhancing the proliferation and differentiation of stromal cells (Tian et al., 2023).

**FIGURE 1:**
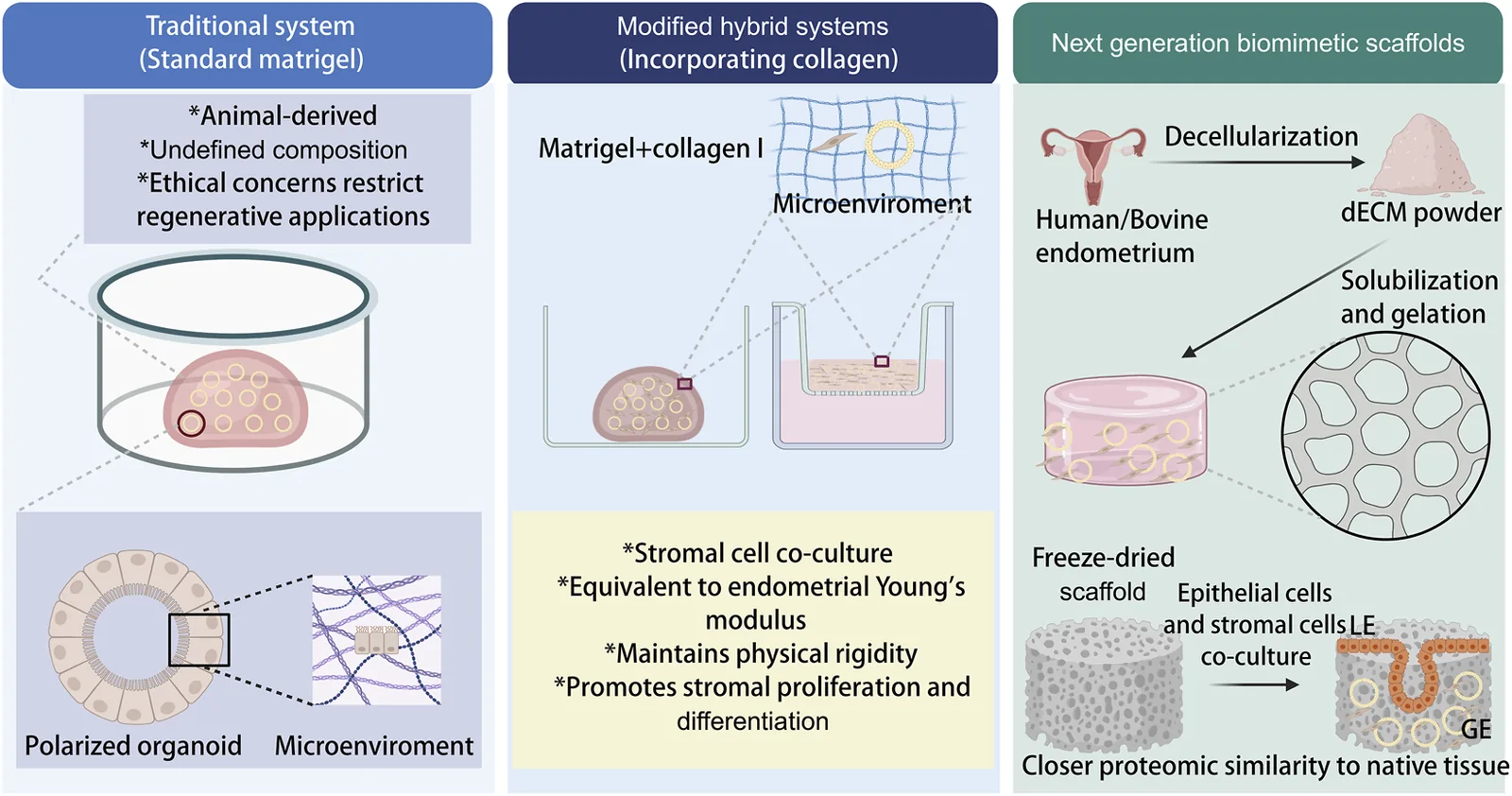
Optimizations in the Extracellular Matrix. The extracellular matrix of endometrial organoids cultured *in vitro* has shifted from traditional Matrigel to novel biomimetic scaffolds.

Another innovative study employed a lyophilization technique where an aqueous slurry of bovine type I collagen was frozen to form ice crystals, which were subsequently removed via sublimation, leaving behind a highly porous structure lined with solid biomaterial (Abbas et al., 2020). Endometrial glandular epithelial and stromal cells were then seeded into this porous scaffold for co-culture and hormonal stimulation. Although this approach successfully advanced 3D structural scaffolding, the matrix notably lacked non-collagenous ECM components, which likely play indispensable roles in orchestrating organoid signal transduction (Figure 1).

More recently, researchers have developed decellularized extracellular matrix hydrogels derived from human and bovine endometrium. These tissue-specific hydrogels support the growth of both mouse and human endometrial organoids, demonstrating proliferative efficiencies comparable to Matrigel while maintaining a proteomic profile that more close to native endometrial tissue (Jamaluddin et al., 2022). ECM is crucial because reciprocal interactions between epithelial and mesenchymal components during development are fundamental in directing tissue morphogenesis and differentiation. Tissue development is not a purely cell-autonomous process, rather it is profoundly instructed by the surrounding microenvironment (Figure 1). Moving forward, the integration of tissue engineering technologies, leveraging 3D scaffolds composed of decellularized matrices, advanced biomaterials, or synthetic polymers, for the construction of endometrial assembloids holds immense promise, particularly for bridging the gap toward clinical applications in regenerative medicine.

## 3 From menstruation to pregnancy: enlightenment from endometrial organoid

### 3.1 Endometrial development and tissue characteristics

The primary functions of the uterus are pregnancy and menstruation (Habiba et al., 2021; Bergmann et al., 2021). In mammals, the uterus originates post-gastrulation from the intermediate mesoderm (Robboy et al., 2017). Around the 8th to 9th week of fetal development, the Müllerian ducts begin to fuse and undergo morphological transformations. In humans, the complete fusion of the Müllerian ducts results in a single uterine cavity. In contrast, incomplete fusion leads to the formation of two separate uterine horns in rodents (Robboy et al., 2017) (Table 1). By the 16th week, the developing human uterus initiates the formation of endometrial glands, which progressively branch and develop complex architectures until birth. This developmental trajectory continues postnatally through puberty, then the uterus undergoes further maturation under the influence of sex steroid hormones in menstrual cycles (Robboy et al., 2017). This contrasts sharply with murine development, where endometrial glands only begin to form postnatally (Kelleher et al., 2019). This developmental divergence likely dictates the species-specific differences observed in the regulation of decidualization (Table 1) and endometrial secretory functions.

**TABLE 1:** Comparative reproductive physiology across representative mammalian species.

Reproductive physiology	Human	Non-human primates	Rodents	Livestock/Ungulates
Representative species	Human	Chimpanzee, macaque, baboon	Mouse, rat	Pig, sheep, cattle
Reproductive cycle	Menstrual	Menstrual	Estrous	Estrous
Uterine anatomy	Simple uterus	Simple uterus	Y-shaped bicornuate uterus	Cornual uterus (pig: long horns; sheep, cattle: short horns)
Implantation type	Interstitial	Interstitial/Superficial (chimpanzee, gorilla: Interstitial; macaque, baboon: Superficial)	Eccentric	Superficial
Placentation type	Hemochorial	Hemochorial	Hemochorial	Epitheliochorial
Decidualization	Spontaneous	Spontaneous	Induced	Induced
Endometrial shedding	Yes (menstruation)	Yes (menstruation)	No (reabsorption)	No (reabsorption)
Suitability for endometrial organoid modeling	Suitable	Highly suitable	Not suitable (for human-specific diseases)	Not suitable (for human-specific diseases)

The precise regulatory mechanisms orchestrating the complex differentiation of the Müllerian ducts into the mature female reproductive system within a restricted developmental window remain incompletely understood. Consequently, Müllerian duct anomalies (MDAs) frequently result in reproductive organ malformations, manifesting clinically as menstrual disorders or infertility (Santana Gonzalez et al., 2021). The prevalence of MDAs rises approximately to 6%–15% among women experiencing infertility and recurrent pregnancy loss (Santana Gonzalez et al., 2021).

To address these knowledge gaps, Venkata et al. successfully established *in vitro* human fetal-derived fallopian tube organoid and endometrial organoid (Venkata et al., 2022). By promoting the *in vitro* differentiation of immature fetal epithelium, this research generated organoids (PAX8+/FOXA2+) with a cellular composition remarkably similar to the adult endometrial simple columnar epithelium, demonstrating that WNT signaling is indispensable for the self-renewal and differentiation of endometrial epithelial cells (Venkata et al., 2022). Even more significantly, when researchers transplanted these fetal-derived endometrial organoids into adult endometrial decellularized scaffolds, the engrafted cells exhibited robust proliferation and successfully differentiated into secretory (PAX8+) and ciliated (AcTUB+) cell subpopulations (Venkata et al., 2022). In a complementary approach, Thomas et al. analyzed scRNA-seq data from neonatal mouse endometrial tissues to map the candidate transcription factors and gene regulatory networks governing the maturation and differentiation of FOXA2+ endometrial glandular epithelium (Spencer et al., 2023). Collectively, these advancements in endometrial organoid research not only provide a unique platform for investigating female reproductive tract anomalies and endometrial regenerative failures but also offer new therapeutic dawn for patients suffering from endometrial injuries.

### 3.2 Regulation of menstrual cycle

During the menstrual cycle, the endometrium undergoes dynamic cyclical changes in both function and morphology. Great apes, Old World monkeys, and some New World monkeys also experience menstrual cycles, characterized by bleeding (menstruation) caused by the shedding of the inner endometrial lining (Catalini and Fedder, 2020). The full thickness of the human endometrium comprises four major components:

1. Epithelial Architecture: Endometrial epithelial cells (EnECs) are the most critical cell population in the tissue. The luminal epithelium (LE) lines the uterine cavity, sheds and regenerates cyclically, and serves as the primary site of initial maternal-embryonic contact (Ye, 2020). The glandular epithelium (GE) is distributed throughout the endometrial layer and transitions from proliferative state to secretory phase under the hormones. During menstruation, epithelial progenitor cells from the GE migrate toward the luminal surface to reconstruct the LE architecture (Bergmann et al., 2021). 2. Stromal Compartment: The endometrial stroma contains numbers of endometrial stromal cells (EnSCs) embedded within a connective tissue extracellular matrix. During the mid-secretory phase, fibroblast-like stromal cells undergo a mesenchymal-epithelial transition (MET) under the progesterone. This transition plays a pivotal role in establishing endometrial receptivity. 3. Vasculature: Hormone receptor expression drives the cyclical changes in endometrial spiral arteries. Elevated progesterone during the secretory phase increases endometrial blood flow to support the WOI. In the absence of pregnancy, progesterone withdrawal triggers spiral artery spasms, leading to decreased blood supply and ischemic necrosis of the stromal tissue, then subsequent menstruation. During pregnancy, the endometrial vasculature transforms into a high-capacity and low-resistance state, driven by the remodeling action of extravillous trophoblasts (EVTs) (Bergmann et al., 2021). 4. Immune Cells: Including natural killer (NK) cells, T cells, B cells, and macrophages—are predominantly localized in the implantation zone. They regulate immune homeostasis, maintain maternal-fetal immune tolerance, and facilitate embryonic invasion. The composition of immune cells fluctuates significantly across the menstrual cycle, with an increased proportion of immunoregulatory cells (such as dNK1, Th2, and M2 cells) being recruited to mediate tolerance during maternal-fetal crosstalk (Garcia-Alonso et al., 2021; Sha et al., 2025).

Notably, most other mammalian species undergo an estrous cycle, during which the endometrium undergoes morphological and functional remodeling (Billhaq et al., 2020). Although cyclical proliferation and regression occur throughout this cycle, sometimes accompanied by reabsorption (Table 1). The endometrial tissue does not shed as it does in menstruating species (Bauersachs and Wolf, 2015; Strassmann, 1996). This physiological distinction is likely attributable to differential expression patterns of hormone receptors in the endometrium across species (Billhaq et al., 2020; Lamy et al., 2025). Consequently, endometrial organoids derived from non-menstruating mammals may not serve as suitable *in vitro* models for studying human decidualization, maternal-fetal interface interactions, or human-specific pregnancy disorders (Table 1). In particular, aspects such as abnormal menstrual bleeding, endometriosis, and shifts in the endometrial immune microenvironment can be relevantly modeled only using human-derived organoids.

Upon the initial endometrial glandular epithelial organoid culture system developed by Turco et al. (2017), researchers sequentially incorporated stromal cells (Cheung et al., 2021; Abbas et al., 2020) and immune cells (Zhang et al., 2023), and refined the luminal epithelial architecture (Tian et al., 2023). A prerequisite for menstruation and the maintenance of pregnancy in humans is the synchronized, cyclical transformation of these four components, orchestrated by the synergistic actions of estrogen and progesterone (Habiba et al., 2021). Organoids have thus emerged as a 3D model for replicating physiological functions *in vitro*.

Under estrogen stimulation *in vitro*, adult progenitor cells derived from primary tissues exhibit high proliferative capacity, with the generation of α-tubulin-positive ciliated cells, and expression of the progesterone receptor (PGR) (Boretto et al., 2017; Cheung et al., 2021; Garcia-Alonso et al., 2021; Turco et al., 2017; Tian et al., 2023). Upon entering the secretory phase, estrogen-induced PGR expression in endometrial organoids actively responds to progesterone, initiating the complex process of decidualization. Although recent studies using endometrial organoids have successfully recapitulated most hallmarks of the WOI (Tian et al., 2023; Garcia-Alonso et al., 2021; Turco et al., 2017), the lack of complete immune and vascular systems precludes the accurate simulation of blood flow regulation and immune tolerance. Nonetheless, these advanced *in vitro* models have provided researchers with a promising avenue for investigating the regulatory mechanisms of endometrial receptivity and implantation, yielding highly encouraging results.

### 3.3 Homeostatic regulation of decidualization: maternal preparation for pregnancy

The term “decidua” originates from Latin, meaning “to fall off” or shed. In females, endometrial decidualization commences approximately 6 days post-ovulation, coinciding with the onset of the WOI. This process involves the morphological and biochemical reprogramming of the four major endometrial components: the decidualization of stromal cells, secretory transformation of endometrial glands, recruitment of uterine natural killer (uNK) cells, and vascular remodeling to support maternal blood supply (Okada et al., 2018).

Some species such as rodents, endometrial decidualization strictly requires induction by embryo implantation or other external stimuli mimicking this process (e.g., intrauterine injection of sesame oil in pseudopregnant mice) (Table 1). The uterine stromal tissue and parietal trophoblast giant cells form the maternal-fetal interface, termed the decidua (Hemberger et al., 2020). Conversely, in humans and some primates, the initiation of decidual differentiation does not require embryonic induction (Okada et al., 2018) (Table 1). Progesterone triggers human endometrial decidualization during the secretory phase. Post-implantation, human chorionic gonadotropin (hCG) maintains progesterone synthesis, allowing decidualization to persist. In non-conception cycles, declining progesterone led to the shedding of the decidua as menstruation.

Progesterone regulates the homeostatic state of endometrial receptivity via classic nuclear receptors (PGR, including PRA and PRB) and PGR ligands (e.g., selective progesterone receptor modulators, SPRMs) (Bagchi et al., 2019). Single-cell transcriptomic atlases during menstrual cycle reveal that receptivity is activated transiently. The transition is abrupt and discontinuous under the key regulatory networks involve HOXA10, FOXO1, HAND2, and various signals and activators of transcription (Wang W. et al., 2020). Upon entering the secretory phase, stromal cells secret factors such as DKK1, BMP2, PD-L1, and WNT4, which further regulate decidual differentiation, including through direct interactions with receptors on the surface of immune cells (Zarnani et al., 2024; Sha et al., 2025). Ultimately, the decidua governs both menstruation and pregnancy, decidualization failure or resistance is associated with a spectrum of reproductive anomalies, including implantation failure, pregnancy loss, and other obstetric complications.

Recent investigations into decidualization mechanisms using endometrial organoids have provided novel insights. *In vitro* decidualized endometrial organoids can be rapidly generated by supplementing cultures with progesterone and cAMP to activate protein kinase A (PKA)-dependent pathways (Cheung et al., 2021; Tian et al., 2023). Within endometrial assembloids, epithelial cells activated by progesterone-induced IHH signaling undergo decidualization through dynamic crosstalk with human pluripotent stem cell-derived endometrial stromal fibroblasts (Cheung et al., 2021). Furthermore, Rawlings et al. utilized organoid models to demonstrate that the rapid senescence phenotype of the decidualized epithelium modulates the secretion of implantation factors, thereby promoting embryo implantation. They also discovered that a tyrosine kinase-dependent stress response dictates the fate of decidual cells, which can subsequently interfere with implantation (Rawlings et al., 2021). Organoids have also functionally validated transcriptomic mappings, confirming that the dynamic balance between WNT and NOTCH signaling acts as a gatekeeper for the WOI during the mid-secretory phase (Wang W. et al., 2020). A novel endometrial assembloid suggests that the dynamic regulation of luminal epithelial cilia also critically impacts the transient opening of the WOI (Tian et al., 2023). Collectively, these achievements illustrate that organoid offer unique innovative potential and distinct advantages for reproductive research compared to animal models.

## 4 Implantation: the dawn of pregnancy

The most profound reproductive divergence across species is the process of embryo implantation (Table 1). This encompasses distinct endometrial preparatory processes—decidualization, variations in trophoblast lineage potential and invasion depth, maternal regulation of the embryo, and the ultimate architecture of the placenta. Based on the anatomical position of the embryo relative to the endometrium, implantation is broadly categorized into three types: centric, superficial, and interstitial implantation (Figure 2).

**FIGURE 2:**
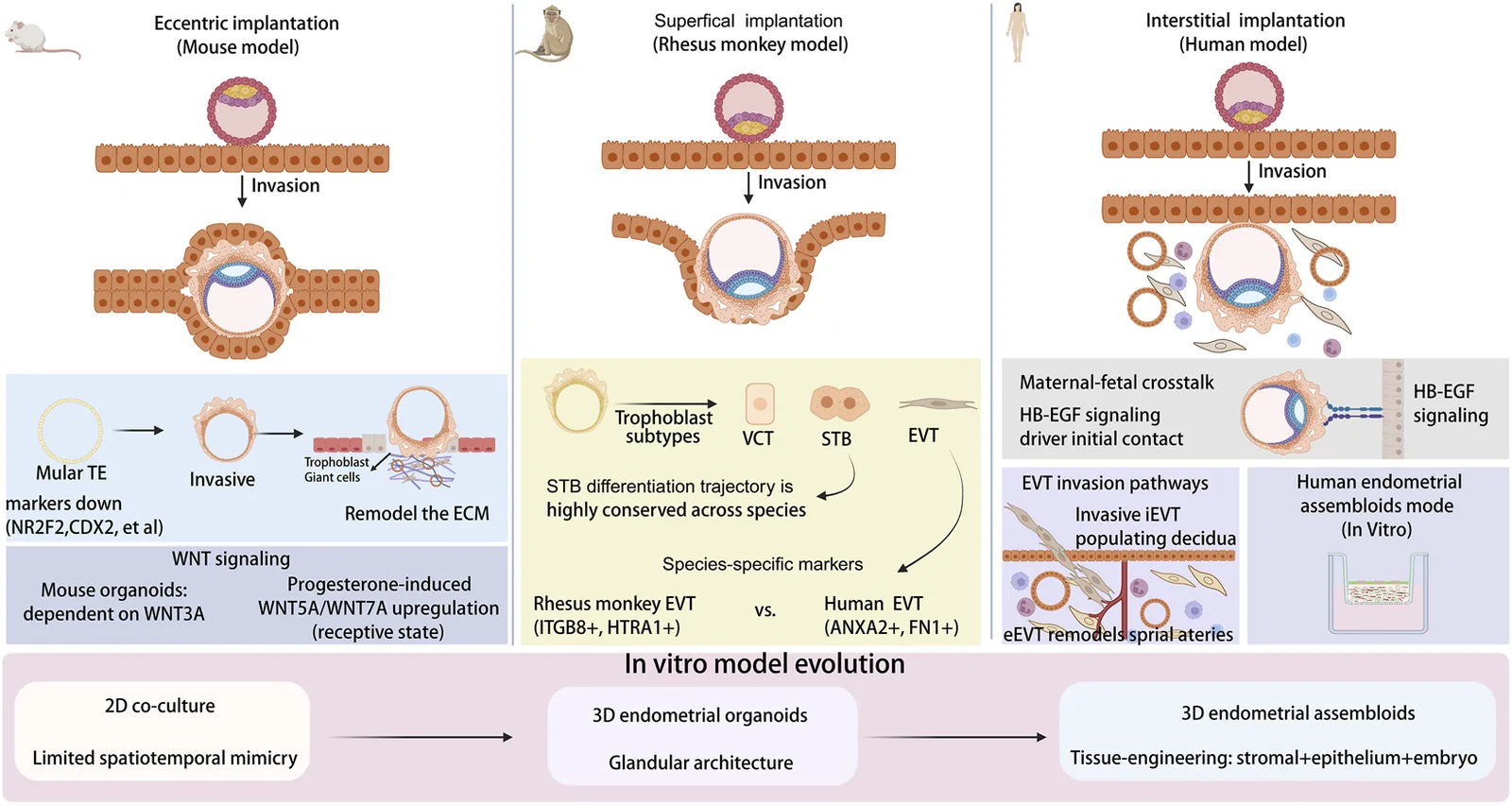
The process of embryo implantation across species. According to the anatomical position of the embryo relative to the endometrium, implantation can be roughly divided into three types: central, superficial, and interstitial implantation (represented by mouse, Rhesus, and human). TE, trophectoderm; ECM, extracellular matrix; VCT, villous cytotrophoblast; STB, syncytiotrophoblast; EVT, extravillous trophoblast; iEVT, interstitial EVT; eEVT, endovascular EVT; HB-EGF, heparin-binding EGF-like growth factor.

### 4.1 Eccentric implantation

Despite the close evolutionary proximity between mice and primates, significant divergence is resulting in marked differences in the critical events at the maternal-fetal interface (Chazaud and Yamanaka, 2016). In murine embryos, the initial lineage segregation relies on the differential activation of the Hippo/YAP signaling pathway between inner and outer blastomeres, driving the expression of the trophectoderm-specific gene CDX2 (Figure 2) (Chazaud and Yamanaka, 2016). Prior to implantation, concurrent with the downregulation of the mural trophectoderm marker CDX2, trophectoderm undergo an epithelial-mesenchymal transition (EMT) (Christodoulou et al., 2019), and differentiate into specialized trophoblast giant cells with the invasive phenotype, that make initial contact with the endometrium.

Following maternal adhesion, murine multinucleated trophoblast giant cells secrete matrix metalloproteinases to remodel the endometrial ECM and partially invade the endometrium. Concurrently, cells at the polar trophectoderm exposed to the uterine cavity maintain proliferation and form the extraembryonic ectoderm, which ultimately expands and differentiates into a component of the murine placental labyrinthine structure (Siriwardena and Boroviak, 2022). The mature mouse placenta is hemochorial, and its labyrinth functions as the primary exchange interface via a complex network of trophoblast-lined sinusoids, where maternal blood directly contacts fetal trophoblasts (Jiang et al., 2023). In contrast, the human hemochorial placenta utilizes chorionic villi as its functional units for exchange (Table 1). Crucially, murine trophoblast invasion is superficial and restricted, contrasting starkly with the deep and extensive trophoblast invasion characteristic of human interstitial implantation (Figure 2). Furthermore, while murine maternal vascular structure and function are altered during pregnancy, this remodeling is primarily mediated directly by uNK cells (Cheng et al., 2023). Whether uNK cells exert identical roles or share molecular mechanisms in humans during the immune response to the semi-allogeneic conceptus remains a compelling question requiring further investigation.

As a widely utilized animal model, mouse offer advantages in husbandry, sample accessibility, reproducible experimental outcomes, and ethical compliance. Consequently, researchers have established mouse endometrial organoids and assembloids to facilitate *in vitro* studies and circumvent the inherent limitations of *in vivo* research. Boretto et al. initially established both human and mouse endometrial glandular organoids, noting that culture conditions were highly conserved across the species, with the notable exception of WNT3A requirements (Boretto et al., 2017). In mouse, the level of WNT dictates the differentiation of the endometrial organoids, highly dependent on WNT concentration (Figure 2). Conversely, because WNT ligands are endogenously expressed in human endometrial cultures, human organoid growth *in vitro* is not reliant on WNT3A supplementation.

WNT signaling, modulated by estrogen and progesterone, is pivotal in regulating the cyclic growth and regression of the human endometrium (Gu et al., 2022; Venkata et al., 2022). In the estrous mouse endometrium, WNT5A and WNT7A are significantly upregulated under progesterone stimulation, marking the receptive state for embryo implantation (Figure 2) (Cha et al., 2014). The human endometrium, however, exhibits profound dynamic complexity. Spatial transcriptomic data indicate that during the mid-secretory phase, the WNT pathway is activated and NOTCH is suppressed in the luminal epithelium, whereas the inverse occurs in the glandular epithelium (Wang W. et al., 2020). This specialized transition preparing for blastocyst implantation has been functionally corroborated *in vitro* using organoid models (Wang W. et al., 2020). This regulatory discordance between the luminal and glandular epithelium is putatively linked to human deep implantation. As part of the implantation zone, the glandular epithelium needs to enter a receptive state to accommodate further embryonic invasion, whereas mouse embryos only invade the luminal and superficial glandular epithelium. Correspondingly, the receptive mouse glandular epithelium does not exhibit the characteristic coiled and distended secretory morphology in humans. Organoid models provide an ideal platform to deeply investigate the differential roles and regulation of the WNT pathway in endometrial homeostasis, remodeling, and implantation across species.

In recent years, considerable progress has been made in establishing mouse organoid and assembloid based *in vitro* models to study embryo implantation. Bondarenko et al. employed 3D geometrically patterned hydrogels to mimic the biomechanical signals of the uterine microenvironment, revealing that integrin-mediated adhesion and Rac1-dependent collective trophoblast migration are essential for implantation and egg cylinder patterning (Bondarenko et al., 2023). An apical-out mouse endometrial organoid containing both epithelial and stromal cells has been established, which upon co-culture with blastocysts recapitulates the full implantation sequence and enables its first *in vitro* visualization (Fujimura et al., 2025). Complementing these endometrial platforms, TBL-blastoids have been shown to implant into mouse uterine and induce decidualization, while Nr1h2-activated blastoids exhibit enhanced implantation competence (Wong et al., 2024). ALI mouse endometrial assembloids have been shown to form a luminal epithelial layer and recapitulate receptive state molecular features (Qiu et al., 2026). Collectively, these mouse models constitute complementary platforms for elucidating the cellular and molecular events of implantation, spanning initial adhesion through trophoblast invasion and decidualization.

### 4.2 Superficial implantation

Superficial implantation (surface attachment), defined by simple contacts or adhesion of the embryo to the endometrial surface, is characteristic of domesticated livestock and lower primates (Table 1) (Figure 2) (Carter and Pijnenborg, 2011). In these livestock species, this implantation mode is linked to a non-invasive epitheliochorial placenta. In this model, the blastocyst trophectoderm penetrates only the endometrial epithelial layer down to the basement membrane, without invading the endometrial stroma, and is often constrained from excessive invasion by a unique epithelial plaque reaction (Bergmann et al., 2022).

In livestock species, including pigs, sheep, and cattle, all of which exhibit superficial implantation, substantial progress has been made in developing *in vitro* models to study fetal-maternal interactions. Hormonally responsive endometrial epithelial organoids have been established in pigs and cattle, demonstrating steroid hormone-induced upregulation of receptors and downstream genes, and supporting embryonic attachment in co-culture systems (Han et al., 2025; Devkota et al., 2026). Porcine endometrial organoid models have been demonstrated to support both embryonic attachment and elongation, recapitulating a key event of superficial implantation for the first time in an *in vitro* system (Saadeldin et al., 2024). In sheep, the first functional endometrial luminal epithelial organoids have been established using an optimized medium containing CHIR99021, Y-27632, SB202190, and EphrinA1 ligand. These organoids exhibit polarized architecture, mucin secretion, and an apical-out polarity (Guo et al., 2026). Functional validation confirmed their capacity to promote blastocyst expansion and trophoblast proliferation, with adhesion enhanced by hormone treatment (Guo et al., 2026). In cattle, a hormonally responsive endometrial organoid model has been developed as a tool for studying endometrial function and peri-implantation communication (Edge et al., 2026). Collectively, these livestock organoid platforms, ranging from epithelial only to multicellular systems and apical-out configurations, provide physiologically relevant *in vitro* systems for dissecting the mechanisms of superficial implantation and early pregnancy establishment in large animal models.

Intriguingly, some primates, including Old World monkeys and great apes, have evolved a true villous hemochorial placenta during pregnancy (Siriwardena and Boroviak, 2022). These species exhibit varying degrees of trophoblast-uterine interactions, encompassing the extent of decidualization, cytotrophoblast formation, interstitial trophoblast invasion into maternal tissues, and the remodeling of uterine spiral arteries (Carter and Pijnenborg, 2011). Recent studies have even documented signs of human-like deep implantation in gorillas and chimpanzees, occasionally accompanied by preeclampsia-like symptoms (Siriwardena and Boroviak, 2022) (Table 1). The diversity of implantation modes among non-human primates carries important evolutionary implications (Table 1). Superficial implantation represents the ancestral state of placental mammals, whereas deep interstitial implantation has evolved independently at least eight times (Siriwardena and Boroviak, 2022). This species-specific variation underscores the need for careful model selection when studying human implantation, as the shallower implantation in macaques may not fully reflect the deep trophoblast invasion characteristic of human pregnancy.

However, the molecular mechanisms underlying these species-specific differences among primates remain elusive, largely due to a paucity of reliable *in vitro* models. A recent groundbreaking study successfully established the first rhesus macaque placental organoid model. By systematically comparing it with human placental organoids using single-cell and single-nucleus RNA sequencing, researchers deeply analyzed the transcriptomic profiles of trophoblast cells, unveiling the divergent molecular regulatory mechanisms distinguishing superficial implantation from interstitial implantation (Caldwell et al., 2025). Rhesus macaque placental organoids successfully differentiated into multiple trophoblast sub-lineages, including cytotrophoblasts (CTBs), syncytiotrophoblasts (STBs), and EVTs. Organoids secreted hormones such as macaque chorionic gonadotropin (mCG) and progesterone, exhibiting a transcriptional profile highly congruous with *in vivo* tissues. The findings revealed that while the STB differentiation trajectory is highly conserved across species, the underlying transcriptional programs possess species specificity. Notably, EVTs exhibited profound species-specific divergence: rhesus EVTs highly expressed genes such as *ITGB8* and *HTRA1*, whereas human EVTs were enriched for *ANXA2* and *FN1*, highlighting the evolutionary divergence in invasive behaviors (Figure 2). The establishment of primate decidual and placental organoids vastly enhances our comprehension of the evolutionary molecular mechanisms governing the placenta as the crucial organ of pregnancy, and offers novel perspectives for the treatment of pregnancy-related complications (Caldwell et al., 2025).

### 4.3 Interstitial implantation

Human embryos undergo interstitial implantation (Table 1), a process stringently orchestrated by a multitude of maternal and fetal factors (Figure 2) (Idelevich and Vilella, 2020). The decidualization plays the central role in the establishment and maintenance of pregnancy. Once embryo implantation initiates, the entire decidualization process intensifies, characterized by the loosening of intercellular junctions within the luminal epithelium, robust secretory activity of the glandular epithelium, continued mesenchymal-epithelial transition (MET) of stromal cells, and the chemotactic recruitment of immune cells to the implantation site (Gellersen and Brosens, 2014).

The initial contact between the embryonic trophectoderm and the endometrial luminal epithelium (LE) is established via sophisticated signaling crosstalk, driven primarily by heparin-binding EGF-like growth factor (HB-EGF) signaling (Figure 2) (Aplin et al., 2017). The apical surface of the LE exhibits increased adhesiveness, marked by the upregulation of cell adhesion molecules (CAMs) including cadherins, integrins, selectins, and mucins. Secreted osteopontin (SPP1) interacts with integrins on both the LE and trophoblast surfaces, bridging the attachment of the embryo to the LE (Xiao et al., 2014). Following this, trophoblast cells proliferate and differentiate into extravillous trophoblasts that invade the decidua. The invasive interstitial EVT (iEVTs) colonizes the decidua and the inner myometrium, while endovascular EVTs (eEVTs) migrates down the lumens of the uterine spiral arteries (Figure 2) (Vento-Tormo et al., 2018). Ultimately, the human embryo fully embeds within the endometrium and forms a villous hemochorial placenta to supply nutrition for subsequent development. Surrounding the invading conceptus, decidual cells provide histotrophic support, establish an immune-tolerant microenvironment, mediate feedback mechanisms to recognize embryo quality, buffer against physiological stressors, and protect the embryo from excessive invasion back into the uterine cavity (Gellersen and Brosens, 2014).

Over the past years, significant progress has been made in developing *in vitro* models that recapitulate human embryo implantation. Endometrial organoid and assembloid-based platforms have emerged as powerful tools for modeling the maternal side of the implantation interface. Rawlings et al. pioneered the transmission of an embryo into an *in vitro* endometrial assembloid (Rawlings et al., 2021) to investigate the impact of the senescence-associated secretory phenotype on embryo implantation. They observed that as the trophectoderm migrated through the ECM, the entire embryo underwent expansive invasion within the complex. This landmark study propelled *in vitro* implantation modeling from 2D-2D (maternal-fetal) systems to highly physiological 3D-3D configurations (Figure 2). Furthermore, by incorporating human embryonic stem cell-derived blastoids into endometrial assembloid, Arima et al. discovered that endometrial epithelial cells were disrupted by the STB. Subsequently, the STB fused with endometrial stromal cells and progressively invaded into the deeper compartments of the assembloid, revealing trophoblast-stromal fusion during endometrial penetration (Shibata et al., 2024). Zhang et al. established a WOI assembloid system that recapitulates pinopodes, cilia, and hormone responsiveness, demonstrated significantly enhanced blastoid adhesion and survival (Zhang et al., 2023). A receptive endometrial scaffold, modeling the luminal, glandular, and stromal compartments, has been established and supports human embryo implantation with 60% efficiency, enabling trophoblast differentiation into primary villi and cytotrophoblastic shell, which are structures critical for placental anchorage (Mole et al., 2026). Similarly, a 3D in-chip implantation platform using bioengineered endometrioids has been shown to recapitulate the complete sequence of apposition, attachment, invasion, and post-implantation development (Li et al., 2026).

These advanced models provide unprecedented vantage points for deciphering embryo implantation and maternal-fetal crosstalk, offering invaluable insights that drive the evolution of reproductive medicine. However, because of the extensive and intricate maternal-fetal interaction, there remains an urgent need for the development of endometrial assembloids that more faithfully replicate *in vivo* anatomical architectures and encompass a complete cellular composition.

## 5 Endometrial organoid technology in female reproductive biomedicine

The uterus is the foundational organ of female reproductive function, and injured endometrium frequently affect reproductive health. More importantly, endometrium disorders lead to adverse pregnancy outcomes. Approximately two-thirds of implantation failures are attributed to endometrial pathologies, functional impairments, or aberrant embryo-endometrial crosstalk (Vento-Tormo et al., 2018). Different *in vitro* models of endometrial organoids have been successfully used to elucidate the mechanisms of certain endometrial diseases and to gain new insights, providing a reliable platform for the establishment of precise clinical treatments (Table 2).

**TABLE 2:** Endometrial organoids for disease modeling.

Reproductive disease	Model and components	New insights	Reference
Endometrial tumors	Endometrial glandular organoids	The morphology of the organoids resembles the primary tumor.	Turco et al. (2017)
	Endometrial glandular organoids	Endometrial cancer-derived organoids accurately capture cancer subtypes, replicate the mutational landscape of the tumors.	Boretto et al. (2019)
	Endometrial glandular organoids	Expression of differentiation markers positively correlates with disease-specific and overall survival of endometrial cancer patients.	Cochrane et al. (2020)
	Endometrial glandular organoids	*Kras* activation drives cellular transformation and epithelial-mesenchymal transition.	Maru et al. (2021)
Endometriosis	Endometrial glandular organoids	Organoids from endometriosis show disease-associated traits and cancer-linked mutations.	Boretto et al. (2019)
	Endometrial epithelial, stromal cells and smooth muscle cells	BMP/WNT signal pathway imbalance promotes adenomyosis.	Xu et al. (2026)
Polycystic ovary syndrome	Endometrial epithelial and stromal cells	Excess androgens in PCOS promoted cell proliferation in endometrial organoids.	Wiwatpanit et al. (2020)
	Endometrial epithelial cells	The suppression of the IL-22-STAT3-IGFBP5 axis is a key contributor to impaired endometrial receptivity in PCOS.	Liao et al. (2026)
Reproductive tract infections	Cervical epithelial organoids	HPV16 E6E7 interferes with *Chlamydia* development and induces persistence.	Koster et al. (2022)
Thin endometrium	Endometrial epithelial organoids and stromal cells	SFRP4+ stromal cells could significantly promote the regeneration of endometrial epithelial glands.	Wu et al. (2022)
Implantation	Epithelial organoids and stromal cells	Senescence phenotype of decidual cells promotes embryo implantation.	Rawlings et al. (2021)
	HUVECs, endometrial epithelial cells and stromal cells	Fusion of syncytiotrophoblasts and stromal cells may occur during early implantation.	Shibata et al. (2024)
	Endometrial epithelial cells and stromal cells	Embryo-maternal interaction is essential for post-implantation human development. Disruption of maternal signals markedly impairs embryonic progression.	Song et al. (2026)

PCOS, polycystic ovary syndrome; HPV, human papillomavirus; HUVECs, human umbilical vein endothelial cells.

### 5.1 Endometrial tumors

Following the establishment of endometrial organoids, several studies have characterized models derived from diverse female donors (Turco et al., 2017; Boretto et al., 2019), comparing the *in vitro* phenotypes, genetic profiles, and hormone responsiveness of normal versus pathological endometrium. RNA sequencing data reveal that organoids derived from endometrial tumors exhibit expression profiles highly concordant with the tumor tissue. Remarkably, even after extensive long-term *in vitro* expansion, organoids retain the majority of the original genetic aberrations, demonstrating high mutational stability. Furthermore, standardized chemotherapeutic drug sensitivity assays indicate that these organoids faithfully recapitulate patient-specific drug responses (Boretto et al., 2019). These findings suggest that tumor-derived organoids serve as optimal platforms for oncology-related drug screening, effectively enhancing the precision of clinical therapeutics. Additionally, distinct subtypes of tumor-derived organoid epithelial cells, including ciliated cells (marked by DYDC2, CTH, FOXJ1, and P73) and secretory cells (marked by MPST),positively correlate with disease specificity and overall survival in endometrial cancer patients (Cochrane et al., 2020). Beyond prognostic value, researchers have also transplanted gene-edited endometrial organoids into nude mice to evaluate oncogenicity, successfully validating the activation mechanisms of Kras in endometrial tumorigenesis (Maru et al., 2021).

### 5.2 Endometriosis

Globally, endometriosis affects approximately 10% of women of reproductive age, with its prevalence surging to 25% among women experiencing infertility (As-Sanie et al., 2025). Although classified as a benign condition, it exhibits malignantly invasive and recurrent behaviors (As-Sanie et al., 2025). Endometriosis impairs fertility on multiple levels. Beyond compromising ovarian function and gamete quality, it disrupts endometrial receptivity by promoting abnormal cellular proliferation and adhesion, localized fibrosis, and profound immune dysregulation, triggering a robust local inflammatory response via the massive recruitment of inflammatory cytokines (As-Sanie et al., 2025). Endometrial organoids can efficiently capture heterogeneity of endometriosis, preserve key characteristics of the tissue, including its genetic background. Organoids also successfully recapitulate disease lesions upon *in vivo* transplantation (Boretto et al., 2019). Establishing patient-derived organoids of endometriosis will be invaluable for deciphering its pathogenesis (Xu et al., 2026). In particular, incorporating immune cells into endometrial organoids in future will provide strategic avenues for identifying novel drug targets, ultimately offering therapeutic options to improve endometrial receptivity and successful pregnancy for infertile patients with endometriosis.

### 5.3 Polycystic ovary syndrome (PCOS)-associated endometrial dysfunction

Polycystic ovary syndrome (PCOS) is a prevalent endocrine disorder associated with significant endometrial dysfunction and poor reproductive outcomes, including reduced implantation and increased miscarriage rates (Wei et al., 2024; Matsuyama et al., 2024). Emerging evidence indicates that women with PCOS, despite pharmacological restoration of ovulation, continue to face significantly lower implantation rates, reduced clinical pregnancy rates, and higher risks of early pregnancy loss compared to women with tubal-factor infertility (Chen et al., 2023). Mechanistically, hyperandrogenism, progesterone resistance, and insulin resistance disrupt epithelial integrity, stromal decidualization, and endometrial receptivity through dysregulated LIF, HOXA10, and inflammatory pathways (Ye et al., 2025; Matsuyama et al., 2024; Kumar et al., 2026; Wang et al., 2023).

Recent endometrial organoid models have recapitulated these abnormalities. PCOS-derived epithelial organoids show increased inflammation (OSMR, ICAM1), reduced size, and impaired hormone responses with decreased LIF and PAEP expression (Luyckx et al., 2025). PCOS-like androgen exposure promotes proliferation and alters gene expression in endometrial organoids (Wiwatpanit et al., 2020). GPX4 deficiency-induced ferroptosis activates TGF-β1/Smad2/3-mediated fibrosis (Ye et al., 2025), and metabolic profiling reveals altered glutamine and TCA cycle metabolism affecting decidualization and embryo attachment (Yang et al., 2026). Li et al. recently revealed the central role of the IL-22-STAT3-IGFBP5 axis in regulating endometrial receptivity in PCOS, and this was validated in endometrial organoids (Liao et al., 2026). In summary, PCOS-associated hormonal imbalances directly impair endometrial receptivity, decidualization, and the immune microenvironment. Endometrial organoids derived from PCOS patients serve as valuable *in vitro* models for elucidating the molecular mechanisms underlying implantation failure and for screening potential therapeutic interventions to improve pregnancy outcomes in this patient population.

### 5.4 Reproductive tract infections and chronic endometritis

Reproductive tract infections are often regarded as a “silent” epidemic, closely linked to gynecological and reproductive complications, frequently culminating in endometritis. Next-generation sequencing (NGS) reveals that CE patients exhibit a disrupted intrauterine microbiome, marked by a decreased proportion of *Lactobacillus*, an increase in non-*Lactobacillus* species, and aberrant microbial metabolism. However, the exact mechanisms driving this microecological dysbiosis and the core pathogenic bacterial consortia remain undefined.

Microbial infections not only inflict structural damage and inflammation but also trigger a cascade of local immune dysregulation. Following pathogen invasion, antigens such as lipopolysaccharide (LPS), stimulate the migration and infiltration of B cells into the endometrial stroma, where they differentiate into plasma cells and secrete immunoglobulins (IgM, IgA, and IgG) (Kitaya and Yasuo, 2010). This severe imbalance in the endometrial immune microenvironment critically disrupts the gestational process, impairing placental angiogenesis, trophoblast invasion, and spiral artery remodeling, ultimately leading to adverse outcomes such as infertility, RIF, and recurrent pregnancy loss (RPL). A recent study utilizing cervical epithelial organoids infected with HPV and *Chlamydia* has provided fresh inspiration, demonstrating that organoid-based microbial infection models create unique cellular microenvironments (Koster et al., 2022). Such approaches offer excellent platforms for deeply investigating infection-related pathogenesis in the reproductive tract and elucidating how microbes dictate host cell fate.

### 5.5 Intrauterine adhesions and thin endometrium

Thin endometrium, whether caused by intrauterine adhesions (Asherman’s syndrome) or other etiologies, represents one of the most intractable endometrial barriers in ART. A thin endometrium is generally regarded to result from mechanical injury or infection to the basal layer, rendering the tissue incapable of normal regeneration under hormonal stimulation (Lv et al., 2022). The precise mechanisms by which injury induces abnormal endometrial cellular proliferation remain obscure, and the lack of effective clinical treatments poses a massive challenge for assistance technology. Currently, adhesiolysis, postoperative IUD placement, low-dose aspirin, and conventional estrogen therapies are common clinical interventions, yet post-treatment recurrence rates can soar to 62.5% (Ma et al., 2021).

While multiple studies have demonstrated that mesenchymal stem cells (MSCs) derived from different sources can effectively repair damaged endometrium in rat and macaque models (Dai et al., 2023; Wang L. et al., 2020), these MSCs are not native to the endometrium. Sourcing cells with stem-like properties directly from endometrial tissue likely represents the most optimal cytotherapeutic approach. A recent study discovered that sFRP4+ stromal cells promote the formation of human endometrial epithelial organoids,and facilitate endometrial regeneration in rats upon transplantation into injury sites (Wu et al., 2022). This paradigm suggests that endometrial organoids can provide a novel cellular source for the regenerative treatment of endometrial injury, thin endometrium, and intrauterine adhesions. By genetically editing stromal cells with stem characteristics and co-culturing them with glandular epithelial organoids to form novel assembloids, subsequent transplantation may stimulate the regeneration of damaged glandular epithelium and repair full-thickness endometrial injuries. This presents highly promising new conceptual frameworks for investigating the mechanisms of endometrial repair and regeneration.

### 5.6 Unexplained RPL, RIF, and obstetric complications

Beyond implantation failures caused by defined uterine pathologies, a substantial population of women experience unexplained RIF and RPL, the molecular mechanisms of which remain elusive (Shaulov et al., 2020; Gellersen and Brosens, 2014). These conditions are thought to stem from a decline in endometrial receptivity or selectivity, frequently accompanied by aberrant decidualization that ultimately compromises fertility (Gellersen and Brosens, 2014). Recent work has demonstrated that lactate promotes endometrial receptivity through H3K18la-mediated upregulation of SLC7A11, driving epithelial-mesenchymal transition and facilitating blastoid adhesion and expansion (Dong et al., 2026). Furthermore, advancements in ART have enabled clinicians to detect pregnancy outcomes earlier, revealing a high incidence of unexplained early implantation failures, clinically termed biochemical pregnancies (Shaulov et al., 2020). This compels us to re-evaluate whether decidualization functions as an innate biological program designed to screen out abnormal embryos. A fine decidual reaction endows the endometrium not merely with passive receptivity for implantation, but with the active selective for viable embryos.

Receptivity manifests as the endometrium’s maximal tolerance for an embryo within a specific spatiotemporal window. Decidualized stromal cells not only provide an optimal microenvironment for trophoblast proliferation but also dynamically mobilize around the embryo to accommodate its expansion, actively participating in the implantation process by encapsulating the blastocyst (Brosens and Gellersen, 2010). In models of endometrial organoid-embryoid interactions have revealed that decidualized stromal cells fuse with syncytiotrophoblasts, progressively losing their original cellular identity, accompanied by a downregulation of *Vimentin* expression (Shibata et al., 2024). The establishment of the interaction models has brought unprecedented clarity to the nuances of maternal-fetal crosstalk, such as how decidual cells auto-modify to facilitate embryonic invasion, events that were largely overlooked in the past.

Implantation is not solely a unilateral embryonic event, mounting evidence indicates the maternal compartment actively screens and embraces the embryo. Decidualized endometrial cells can act as embryonic biosensors. Concurrently, both human and murine embryos can signal their implantation competency by secreting serine proteases that cleave and activate epithelial Na^+^ channels on the apical membrane of luminal endometrial epithelial cells (Ruan et al., 2012), underscoring the critical role of the luminal epithelium in transducing embryo-derived implantation initiation signals. Accordingly, the construction of endometrial assembloids comprising both luminal epithelium and decidualized stroma represents a major breakthrough. When a physiologically functional, polarized luminal epithelium responds to progesterone by exhibiting reduced ciliogenesis, altered hormone receptor profiles, and enhanced receptivity gene expression, it acquires the receptivity to accept an embryo (Tian et al., 2023). Simultaneously, decidua cultivate an accommodating microenvironment for further embryonic invasion. Zhao and colleagues develop a 3 D co-culture system of human embryos and endometrial organoids that mirrors post implantation, revealing key signaling events at the maternal embryonic interface relevant to early pregnancy success (Song et al., 2026).

We need further elucidate how the embryo selects a singular implantation site across the “vast” expanse of the luminal epithelium—is this mere coincidence or a pre-programmed design? Do non-selected sites harbor adverse factors unsuitable for implantation? Do RIF patients undergoing ART possess inherent functional errors in their luminal epithelium that preclude embryo anchoring? How do trophoblasts initiate crosstalk with stromal cells upon breaching the luminal epithelium? And how do stromal cells discern embryo quality during early contact to dictate whether to permit further invasion? These scientific questions can now be rigorously addressed by leveraging the maternal-fetal interface models constructed from endometrial organoids.

Another characteristic of embryo implantation is the highly complexity of immune tolerance, involving the recruitment and proliferation of diverse immunomodulatory cells, the expression of myriad regulatory molecules and cytokines, and the eventual formation of the placenta, which itself exerts profound immunoregulatory functions (Moffett and Shreeve, 2023). It is generally accepted that uNK cells primarily function by regulating EVT invasion, yet the exact mechanistic pathways remain obscured (Park et al., 2022). Although EVTs must remodel maternal arteries to ensure a successful pregnancy, this behavior must be exquisitely regulated to mitigate the risks of over-invasion (Moffett and Shreeve, 2023). The interactions between various combinations of maternal killer cell immunoglobulin-like receptors (KIRs, including both activating and inhibitory subtypes) expressed by uNK cells and HLA-C variants expressed by fetal EVTs are strongly implicated in placentation disorders, subsequently altering the cytokine and chemokine profiles secreted by EVTs (Moffett and Shreeve, 2023). Severe inhibition of uNK cells results in shallow trophoblast invasion, which is associated with adverse pregnancy outcomes such as low birth weight, increased risk of preeclampsia, and recurrent pregnancy loss, likely driven by insufficient maternal vascular remodeling and defective placentation (Witt et al., 2002). Conversely, reduced uNK cell inhibition, accompanied by increased secretion of cytokines such as CCL4, XCL1, and GM-CSF, correlates with placenta accreta, placenta increta, and an increased frequency of fetal macrosomia (Moffett and Colucci, 2015). Elevated birth weight can precipitate labor abnormalities, shoulder dystocia, maternal trauma, and postpartum hemorrhage, severely jeopardizing both maternal and fetal safety.

How the phenotype and functionality of EVTs incrementally shift as they migrate deeper into the decidua under the regulatory influence of maternal endometrial cells, and how EVT cells so precisely halt their invasion to fuse and form placental bed giant cells within the myometrium—requires extensive further investigation. Endometrial organoids accurately simulate the 3D architecture and physiological functions of the tissue, and particularly when integrating tissue-resident immune cell populations, they faithfully recapitulate the cellular composition and transcriptomic hallmarks of the window of implantation (Zhang et al., 2023). Coupling these advanced models with dynamic visual monitoring and spatial transcriptomics will profoundly enhance our understanding of how maternal immune cells exert precise biological and functional control over the embryonic trophoblast, dictate placental development, and ultimately unravel the underlying regulatory mechanisms of related gestational diseases.

## 6 Interdisciplinary collaboration opportunities for endometrial organoids in maternal-fetal interface research

### 6.1 Engineering disciplines

Organoid models present abundant opportunities for interdisciplinary collaboration in endometrial function research. For instance, integrating biological research with engineering disciplines to co-develop advanced culture technologies and microfluidic devices can significantly enhance the quality and physiological relevance of organoid models (Figure 3). Organoids intrinsically mimic the principles of tissue development and regeneration, where continuous, localized microenvironmental shifts induce the emergence of complex cellular patterns and orchestrate structural morphogenesis. However, the structural formation in traditional glandular organoid cultures is largely stochastic, often resulting in a heterogeneous mixture of columnar and squamous-like glandular structures (Turco et al., 2017; Tian et al., 2023). Achieving precise, spatiotemporal control over biochemical and biophysical condition could direct organoids to form truly biomimetic physiological architectures. This includes engineering chemical signal gradients, such as growth factors and integrins, alongside the manipulation of biophysical parameters like spatial geometry and fluid dynamics (Fukuda et al., 2014). The combination of microfluidics and photochemistry enables the orchestrated control of signaling molecules, dictating their concentration gradients, exposure durations, and spatial distributions (Figure 3). For example, one study successfully utilized a combination of organ-on-a-chip design and microfluidics to exert controlled induction over BMP and WNT signaling pathways, driving the generation of specific structures during ectoderm and amnion development (Zheng et al., 2019).

**FIGURE 3:**
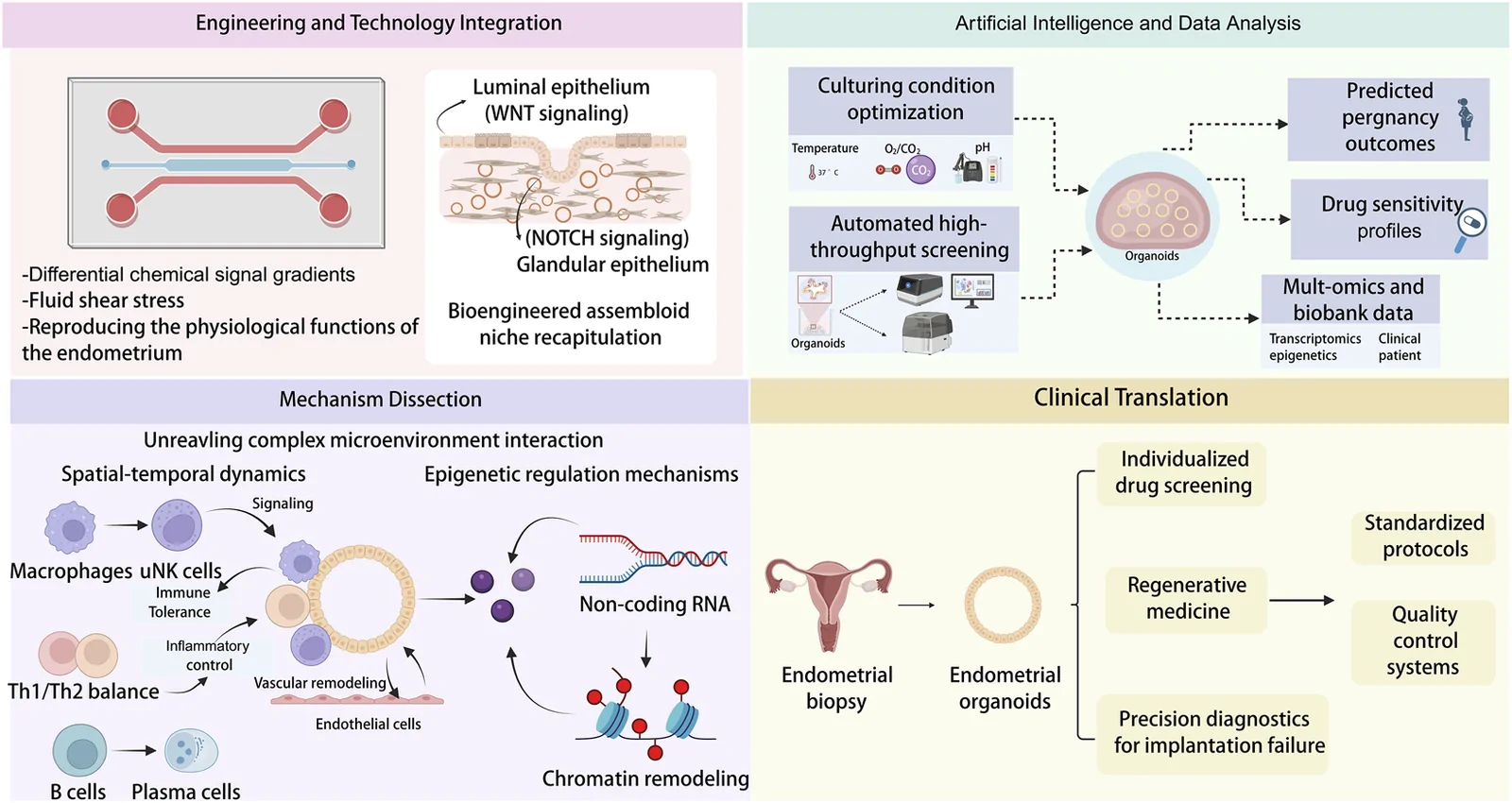
Future perspectives of endometrial organoid. Organoid models present abundant opportunities for interdisciplinary collaboration in endometrial function research (including engineering disciplines and artificial intelligence), and novel perspectives for researchers in reproductive biology.

Currently, a prominent structural defect in endometrial organoids is the anatomical discontinuity between the glandular and luminal epithelium, which diverges starkly from native tissue architecture and likely impact recapitulation of endometrial physiology. *In vivo*, nutrient and growth factor rich secretions produced by the glandular epithelium are transported through the glandular lumen to the luminal epithelium, establishing a highly specialized microenvironment. This is functionally analogous to the crypt-villus architecture of the small intestine. Leveraging bioengineering technologies to reconstruct these invaginated glandular structures within endometrial organoids may profoundly deepen our understanding of endometrial stem/progenitor cell niches, tissue regenerative capacity, microbiome interactions, and the precise regulatory mechanisms governing embryo implantation.

### 6.2 Artificial intelligence

While endometrial organoids hold immense translational promise across disease modeling, drug screening, and personalized ART therapeutics, substantial challenges remain regarding the complexity of their construction and the inherent difficulties of large-scale data analysis. The integration of endometrial organoids with artificial intelligence (AI) and big data analytics offers a powerful solution (Figure 3). This synergy can not only rapidly optimize construction protocols and streamline complex multi-omics data analysis but also analysis the massive datasets generated by organoid models to uncover latent biological insights.

*In vitro* culture conditions are variable, such as temperature, oxygen tension, pH, and growth factor concentrations (Figure 3). They exert profound effects on cellular behaviors, dictating trajectories of proliferation and differentiation (Tian et al., 2023; Boretto et al., 2017). Optimizing these parameters is critical for successful organoid derivation. Machine learning (ML) algorithms can fine-tune these culture conditions, dramatically improving the efficiency and reproducibility of organoids construction. Furthermore, developing novel cell culture protocols is traditionally a labor-intensive, error-prone process. A pioneering study by Kanda et al. utilized an integrated robotic and AI system to autonomously adjust culture protocols, successfully generating specific, highly consistent cell types (Kanda et al., 2022). Similarly, ML can meta-analyze vast repositories of published experimental data to identify the most efficacious growth factor cocktails, establishing optimal protocols for promoting cell growth and organoid differentiation (Gjorevski et al., 2016).

During the organoid generation process, massive volumes of structural and morphological data, including size, shape, architectural complexity, and high-resolution confocal imaging are generated. ML excels at processing these datasets accurately and efficiently, facilitating precise evaluations of organoid functionality. Recent data from the World Health Organization (WHO) indicates that up to one in six couples globally experience infertility (World Health Organisation Guideline Development Group for Infertility et al., 2025). In response, researchers are actively striving to establish comprehensive endometrial organoid biobanks derived from them (Figure 3). Traditional methodologies for extracting and analyzing data from such massive repositories often pose insurmountable challenges. AI, however, is uniquely equipped to interrogate these expansive datasets, identifying hidden pathogenic patterns and correlations that could yield revolutionary discoveries. Moreover, by integrating AI with patient-specific organoid multi-omics data, clinicians could accurately assess the heterogeneity of endometrial receptivity and predict individualized ART outcomes.

### 6.3 Materials science

Finally, ongoing advancements in materials science will undoubtedly provide superior biomaterials tailored to support organoid growth, further optimizing the culture microenvironment (Figure 1). Bridging fundamental basic research with clinical medicine will accelerate the transformation of organoid to the application. Concurrently, the active involvement of ethical and legal disciplines is absolutely paramount. Proactively addressing the bioethical and regulatory frameworks surrounding organoid research will safeguard and facilitate their clinical translation. Ultimately, robust interdisciplinary collaboration will propel the rapid evolution of organoid in endometrial function research, delivering comprehensive, transformative solutions for medical challenges related to reproductive medicine.

## 7 Summary

As a revolutionary *in vitro* model developed in recent years, endometrial organoids have demonstrated immense potential in female reproductive medicine, propelling groundbreaking advancements. By precisely reconstructing cellular components, endometrial assembloids provide an unprecedented dynamic observation platform and mechanistic analytical tool for exploring key biological events, including endometrial development and functional regulation, the pathophysiology and treatment of endometrial diseases, and the microenvironmental regulation at the maternal-fetal interface.

Endometrial organoids derived from tissue or menstrual blood can successfully simulate the physiological processes of cyclical proliferation, secretion, and regeneration, offering a reliable *in vitro* platform to elucidate the molecular networks governing uterine development and the establishment of endometrial receptivity mediated by estrogen and progesterone. Concurrently, patient-derived organoids enable the precise recapitulation of dysfunctional endometrial phenotypes under pathological states, paving novel pathways for deciphering disease pathogenesis and screening targeted therapeutics. Crucially, numerous studies have confirmed that endometrial organoids possessing adhesion and barrier functions can accurately mimic the histological and transcriptomic characteristics of the WOI. Their successful application in investigating the maternal-fetal interface microenvironment has significantly advanced our understanding of human implantation mechanisms, providing fresh therapeutic insights for personalized medicine in infertility and RPL (Figure 3). These achievements not only overcome the inherent limitations of traditional two-dimensional cultures in replicating the 3D endometrial microenvironment but also construct a vital bridge between basic research and clinical translation, laying a crucial foundation for innovative research into endometrial function.

### 7.1 Current challenges and limitations disciplines

Nevertheless, the field still confronts a series of substantial challenges. Under physiological conditions, the endometrium undergoes dynamic and continuous changes in response to the cyclic fluctuations of ovarian hormones. By contrast, current organoid and assembloid cultures are typically maintained under static, constant hormone concentrations. This fundamental difference in culture systems substantially limits the utility of these models for studying hormone-related disorders such as abnormal uterine bleeding and functional regulatory disturbances, and will require future integration of microfluidic perfusion or hormone gradient generators to recapitulate the physiological hormone dynamics. Additionally, these systems are also deprived of native biophysical forces, including peristalsis, fluid shear stress, and matrix stiffness dynamics, which restricts research on endometrial fibrotic pathologies and the physical regulatory mechanisms underlying implantation. Moreover, current organoid models still lack a neuro-innervation system, rendering them incapable of recapitulating the neural components underlying endometriosis-associated pain or primary dysmenorrhea *in vitro*. Another critical limitation is that current endometrial assembloids largely fail to reconstitute the complete vascular networks and immune microenvironment of the endometrium. Consequently, their simulation of complex physiological processes such as the establishment of endometrial receptivity and embryo implantation remains insufficiently precise.

Additionally, organoids are susceptible to phenotypic drift during long-term culture, which compromises the stability and reproducibility of experimental results and limits their ability to fully recapitulate intercellular crosstalk and signaling networks. Mechanistically, organoid-based investigations into the epigenetic regulation of endometrial stem cell maintenance and differentiation remain superficial. In particular, studies linking regulatory networks like non-coding RNAs and chromatin remodeling to endometrial dysfunction are still in their infancy. Furthermore, broader discussions are required at the translational medicine level, particularly concerning the ethical compatibility and technical stability of co-culturing endometrial organoids with human embryos, which remains a critical barrier for achieving functional implantation research. Further exploration of the pathways to translate organoid into personalized clinical diagnostics and therapeutics, alongside the establishment of standardized construction protocols and quality evaluation systems, is essential to facilitate their practical application in personalized drug sensitivity testing and regenerative medicine.

### 7.2 Future perspectives

Looking ahead, addressing these barriers through technological integration and the enhancement of organoids complexity will dictate the primary developmental trajectory of the field. We need to further construct the organoid with precision engineering, progressing toward multi-lineage “assembloids” or “organs-on-a-chip” that integrate key components such as vascular endothelial cells and immune cells to construct holistic “organoid-microenvironment” composite models. Further integration of technologies such as spatiotemporal transcriptomics and live-cell imaging, coupled with AI for the mining and analysis of big data, will enable a more precise recapitulation of the implantation microenvironment. This includes deciphering the complex and meticulously regulated local endometrial immune microenvironment: how macrophages and uNK cells mediate immune tolerance and interact with vascular endothelial cells to drive gestational remodeling, how the Th1/Th2 balance is orchestrated to regulate the pro- and anti-inflammatory equilibrium, facilitating embryo implantation and pregnancy maintenance, and how rare but indispensable B cells migrate from the basal layer to the stroma upon pathogenic stimulation, differentiating into plasma cells to exert protective functions.

Concurrently, there should be a strong focus on technological innovation and multidisciplinary convergence. Developing automated, high-throughput organoid culture and screening platforms driven by AI, and establishing disease-specific biobanks utilizing patient-derived organoids, will allow for the systematic profiling of endometrial organoid heterogeneity. Such platforms will be instrumental in uncovering the epigenetic regulatory mechanisms in the endometrium of patients with unexplained infertility or RPL, identifying key regulatory factors and signaling pathways (Figure 3). This will catalyze breakthrough advancements in the treatment of reproductive disorders and propel the development of precision diagnostics and personalized intervention strategies for implantation failures. Finally, the establishment of standardized protocols for the derivation and quality control of endometrial organoids will rapidly accelerate their clinical translation (Figure 3). This will facilitate organoid-based personalized drug screening for endometrial pathologies, ensure the efficacy and safety of regenerative medical therapies, and ultimately drive the technology from basic research into robust clinical practice.

In summary, as culture technologies continue to optimize and research methodologies continuously innovate, endometrial organoids will play an increasingly pivotal role in unraveling the regulatory mechanisms of endometrial function. They hold the profound promise of delivering revolutionary breakthroughs in the prevention and treatment of major reproductive health disorders, such as infertility and endometriosis, thereby empowering the advancement of reproductive medicine.
